# Supplementary material for: Microfluidic-Assisted Fabrication of Dual-Coated pH-Sensitive Mesoporous Silica Nanoparticles for Protein Delivery
Source: Biosensors (Basel). 2022 Mar 18;12(3):181. doi: 10.3390/bios12030181 (PMC8946851; doi:10.3390/bios12030181)
Supplement: Supplementary file 1 [file biosensors-12-00181-s001.zip › biosensors-1628009-supplementary.pdf]

biosensors

Supplementary

Microfluidic-assisted fabrication of dual-coated pH-sensitive mesoporous silica nanoparticles for protein delivery

Berrin Küçüktürkmen*[1,2], Wali Inam[1], Fadak Howaili[1], Mariam Gouda[1], Neeraj Prabhakar[1] , Hongbo Zhang*[1,3] and Jessica M. Rosenholm[1]

1. Results and Discussion

1.1. Loading Lysozyme into MSNs

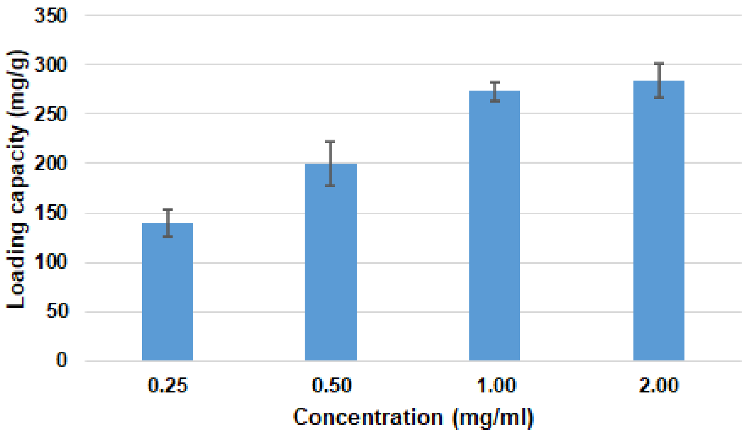

Figure S1. The loading capacities of MSNs.

1.2. In Vitro Lysozyme Release

In vitro lysozyme release studies were performed to determine the release of the drug in neutral pH conditions after the first PDDMA polymer coating process, and to answer the question of whether it is released under acidic conditions after the second SpAcDEX coating. For comparison, release of the lysozyme was also performed before coating processes. The release profiles were compared in pairs to calculate the factor f2, according to *Equation S1*. The factor values for f2 were found to be 33.82, 40.92, and 32.95 for the comparison of release profiles as follows: *MSNs without coating: PDDMA@MSN*; *MSNs without coating: SpAcDEX-coated PDDMA@MSN;* and *PDDMA@MSN: SpAcDEX-coated PDDMA@MSN*, respectively. Finding the values of f factors between 0 and 50 showed that there was a significant difference between the profiles. As seen in Figure S2, lysozyme was released in pH 7.4 phosphate buffer in a sustained manner as almost 45% was released after 4 hours and 86% was released after 18 hours of incubation. After the first coating with PDDMA, the release slowed down as almost 22% was released after 4 hours and 86% was released after 24 hours of incubation. On the other hand, in acidic pH, lysozyme is rapidly released from the particles that were also coated with a second SpAcDEX layer. After 6 hours of incubation, 79% of the lysozyme was released (Figure S1). This provided the desired release mechanism, since cancer cells are known to acidify their environment.

$$f2 = 50 \cdot \log\left\{\left[1 + \frac{1}{n}\sum_{t=1}^{n}(R_t - T_t)^2\right]^{-0.5} \times 100\right\}$$

(Equation S1)

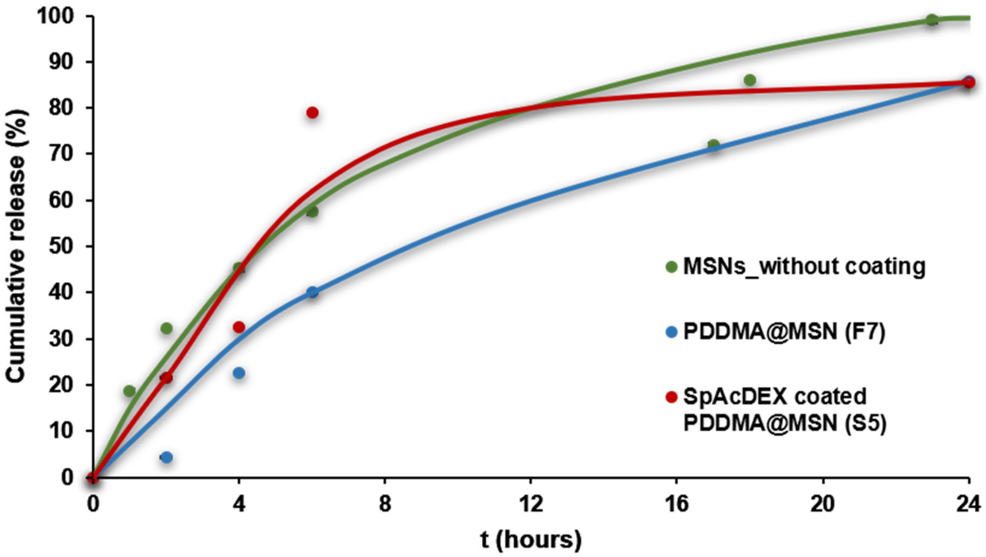

Figure S2. In vitro release of lysozyme from MSNs without coating and PDDMA@MSN (in PBS buffer pH 7.4 at 37°C), and SpAcDEX-coated PDDMA@MSN (in acetate buffer pH 5 at 37°C). Lines are a visual aid.

To study the drug release kinetics, the experimental data were fit to four kinetic models, including zero-order, first-order, Higuchi, and Korsmeyer–Peppas equations. Considering the highest r^2 values, in vitro release of lysozyme from MSNs without coating, PDDMA@MSN and SpAcDEX-coated PDDMA@MSN has shown compliance mainly with the Higuchi ($r^2 = 0.973$, 0.991 and 0.796, respectively) and Korsmeyer–Peppas kinetics ($r^2 = 0.965$, 0.995, and 0.744, respectively) (Table S1). Compliance with Higuchi kinetics indicated that lysozyme, a hydrophilic drug, was primarily released by diffusion from the porous structure of MSNs. The n values between 0.45 and 0.89 in Korsmeyer–Peppas kinetics for these formulations showed that both diffusion and erosion were effective in lysozyme release.

Table S1. Results of analyses of in vitro release kinetics.

**Kinetics**		**MSNs without coating**	**PDDMA@MSN**	**SpAcDEX-coated PDDMA@MSN**
Zero-order	r^2	0.886	0.840	0.615
	k_0	3.540	3.440	3.018
First-order	r^2	0.780	0.995	0.710
	K_1	0.148	0.196	0.075
Higuchi	r^2	0.973	0.991	0.796
	K_h	19.36	20.12	18.49
Korsmeyer–Peppas	r^2	0.965	0.995	0.744
	n	0.481	0.451	0.559

1.3. Cytotoxicity

Cell studies were performed using HeLa cells to evaluate the cytocompatibility of the SpAcDEX-coated MSNs using concentrations ranging from 1 to 50 µg/ml. Our results

showed that the cell viability was higher than 80% after exposure to MSNs at a concentration of 1 μg/ml for 72 h. On the other hand, a decrease in cell viability was observed with increasing concentrations of 10, 20, and 50 μg/ml of MSNs (Figure S3). Due to the highly cationic nature of SpAcDEX and the presence of oligoamine spermine, the positively charged nanoparticle surface easily interacts with the negatively charged cell membrane, promoting high cell–nanoparticle interactions and internalization, resulting in dose-dependent decrease in cell viability [1]. Here we note that the SpAcDEX coating is not meant to be a coating as such for final use but is mainly used for its ample possibilities for further functionalization given its abundance of amine groups. Once the amine groups are consumed for functionalization, the high positive-charge density is also reduced, which, in turn, reduces the cytotoxicity[2]. This phenomenon was observed for PEI, the most cationic polymer available, which, as such, was also known for its high cytotoxicity in multiple earlier studies when used as a coating for MSN by us and others [3].

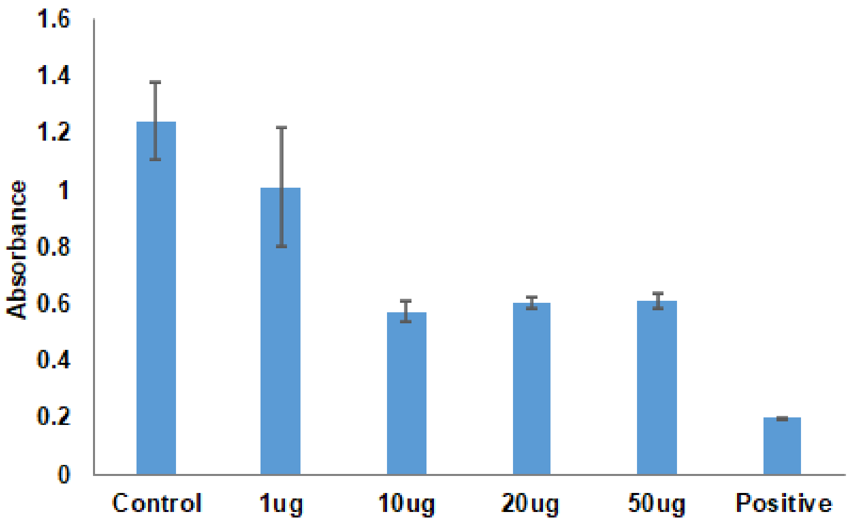

Figure S3. Cell viability of SpAcDEX-coated PDDMA@MSNs performed with WST-1 cell proliferation assay.

2. Materials and Methods

2.1. In Vitro Lysozyme Release

Lysozyme loaded particles were dispersed in 1 ml of PBS buffer (pH 7.4) and samples were shaken in a water bath shaker at 200 rpm at 37°C. At predetermined time intervals, tubes were centrifuged at 13000 rpm for 5 minutes, separating the particles from the continuous phase. The supernatant, which contains the released lysozyme was subsequently analyzed and 1 ml of fresh buffer replaced the withdrawn supernatant. The analysis was performed by using the UV-Vis spectrophotometer, NanoDrop 2000c (Thermo Scientific™). The concentration was calculated based on a standard calibration curve (Figure S4). After a second coating of lysozyme-loaded particles with SpAcDEX, in vitro release was performed in pH 5 acetate buffer.

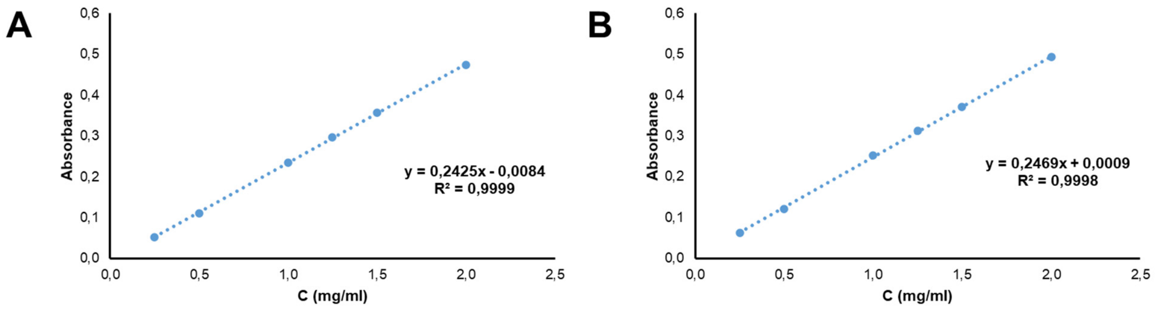

Figure S4. Calibration of lysozyme in PBS buffer pH 7.4 (A) and acetate buffer pH 5 (B).

2.2. Cytotoxicity

The cytotoxicity of PDDMA@MSNs coated with SpAcDEX was evaluated using WST-1 cell viability assay (Roche Diagnostics, Mannheim, Germany). A total of 20,000 HeLa cells per well were grown in a 24-well plate in DMEM (10% fetal calf serum (FCS), 1% amino acids, and 1% penicillin–streptomycin), and incubated overnight to adhere. Then, nanoparticles were sonicated for 2 min and added to 1 mL of prewarmed (37°C) growth medium at four different increasing concentrations of 1 ug/ml, 10ug/ml, 20 ug/ml, and 50 ug/ml. The growth media of the cells in 96-well plates were replaced with media containing nanoparticles. DMSO (Dimethyl sulfoxide) was added at a concentration of 10% as a positive control, whereas negative control cells were untreated (pure cell media only). After incubating the particles for 72 h at 37°C and 5% CO_2, 25 µL of WST-1 cell proliferation reagent (Roche Diagnostics) was added to the wells, and the plate was again allowed to incubate for 2 h at 37°C, 5% CO_2. After the incubation period, the absorbance was read at 430 nm by Varioskan microplate reader (Thermo Scientific™, The U.S.). The number of viable cells was correlated with the observed absorbance from each individual sample.

References

1. Ferreira MPA, Talman V, Torrieri G, Liu D, Marques G, Moslova K, et al. Dual-Drug Delivery Using Dextran-Functionalized Nanoparticles Targeting Cardiac Fibroblasts for Cellular Reprogramming. *Adv. Funct. Materials*. **2018**;*28*(15):1705134.
2. Zhang H, Liu D, Wang L, Liu Z, Wu R, Janoniene A, et al. Microfluidic Encapsulation of Prickly Zinc-Doped Copper Oxide Nanoparticles with VD1142 Modified Spermine Acetalated Dextran for Efficient Cancer Therapy. *Adv. Healthc. Mater*. **2017**;*6*(11).
3. Desai D, Karaman DS, Prabhakar N, Tadayon S, Duchanoy A, Toivola DM, et al. Design considerations for mesoporous silica nanoparticulate systems in facilitating biomedical applications. *Open Mater. Sciences*. **2014**;*1*(1).
